# Supplementary material for: Rab GTPases and phosphoinositides fine-tune SNAREs dependent targeting specificity of intracellular vesicle traffic
Source: Nat Commun. 2024 Mar 20;15:2508. doi: 10.1038/s41467-024-46678-x (PMC10954720; doi:10.1038/s41467-024-46678-x)
Supplement: Supplementary file 1 — Supplementary Information [file 41467_2024_46678_MOESM1_ESM.pdf]

Supplementary materials for

Rab GTPases and phosphoinositides fine-tune SNAREs dependent targeting specificity of intracellular vesicle traffic

Seiichi Koike and Reinhard Jahn

This file includes:

Figure S1-S6 and legends

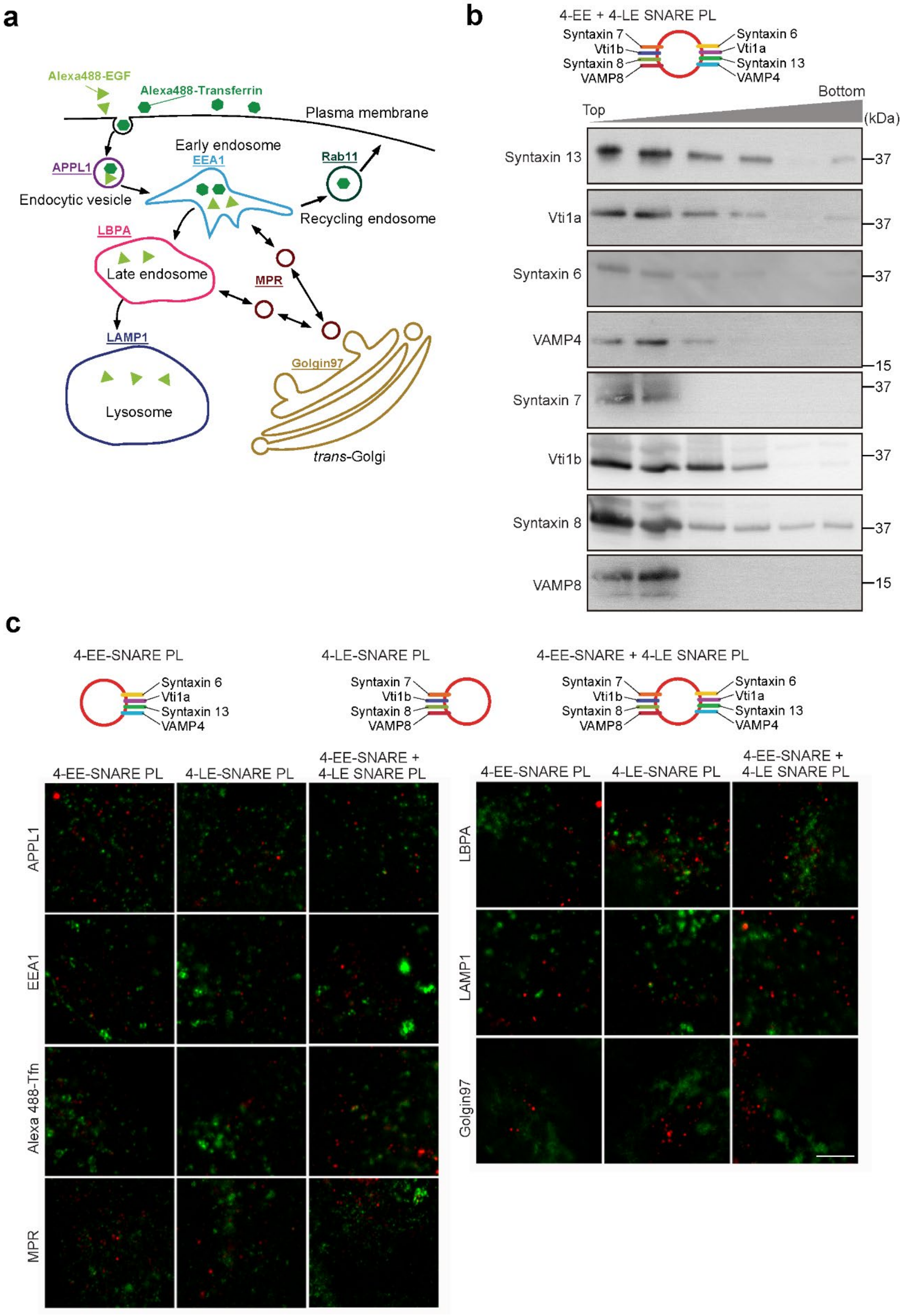
a
Alexa488-EGF
Alexa488-Transferrin
Plasma membrane
APPL1
Endocytic vesicle
Early endosome
EEA1
Rab11
Recycling endosome
LBPA
Late endosome
MPR
LAMP1
Lysosome
Golgin97
trans-Golgi
b
4-EE + 4-LE SNARE PL
Syntaxin 7
Vti1b
Syntaxin 8
VAMP8
Syntaxin 6
Vti1a
Syntaxin 13
VAMP4
Top
Bottom
(kDa)
Syntaxin 13
Vti1a
Syntaxin 6
VAMP4
Syntaxin 7
Vti1b
Syntaxin 8
VAMP8
37
37
37
15
37
37
37
15
c
4-EE-SNARE PL
4-LE-SNARE PL
4-EE-SNARE + 4-LE SNARE PL
4-EE-SNARE PL
4-LE-SNARE PL
4-EE-SNARE + 4-LE SNARE PL
APPL1
EEA1
Alexa 488-Tfn
MPR
LBPA
LAMP1
Golgin97

Supplementary Figure S1

(a) Schematic overview showing the organelle-specific markers used in this study. Abbreviations are given in the text.

(b) Analysis of 4-EE + 4-LE-SNARE liposomes by flotation density gradient reveals efficient incorporation of the SNARE proteins. Proteoliposomes were loaded at the bottom in 80% Nycodenz and overlaid with a discontinuous Nycodenz gradient of decreasing density. During centrifugation, membranes such as proteoliposomes migrate to the top whereas non-incorporated proteins remain at the bottom. Fractions recovered from the gradient were analyzed by SDS/PAGE and immunoblotting for the SNARE proteins.

(c) Related to Fig. 1a. Representative confocal microscopy images of HeLa cells fixed 5 min after injection of 4-EE + 4-LE-SNARE liposomes labeled by Rhodamine-PE. Cells were immunolabeled using organelle-specific markers and subsequently detected with Alexa Fluor 488-labeled secondary antibodies or internalized Alexa Fluor 488-labeled Transferrin (Tfn). Scale bar, 5 µm.

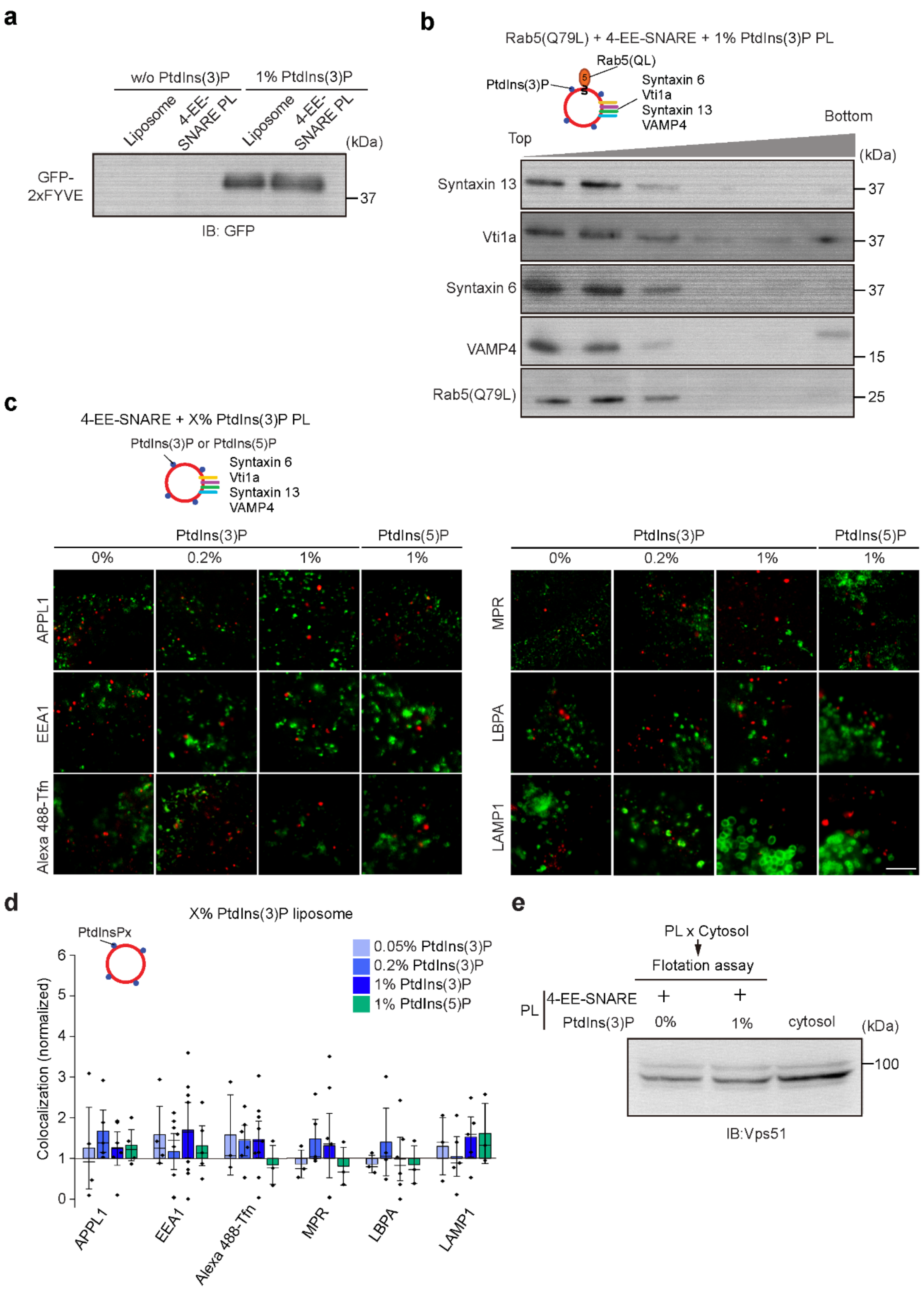

Supplementary Figure S2

(a) GFP-2xFYVE protein binding to (proteo)liposomes, used as a reporter for the presence of

PtdIns(3)P, reveals efficient membrane incorporation of PtdIns(3)P upon reconstitution. A cytosol fraction obtained from HeLa cells overexpressing GFP-2xFYVE was incubated with versions of liposomes (endosomal membrane lipids) that contained, in addition, the four early endosomal SNARE proteins (4-EE-SNAREs), 1% PtdIns(3)P, or both. Incubation was carried out at 37℃ for 30 min. Thereafter, membranes were separated by flotation gradient centrifugation, and the binding of the GFP-2xFYVE reporter protein was detected by immunoblotting (IB) with a GFP antibody.

(b) Flotation gradients of liposomes containing endosomal lipids and reconstituted with SNAREs, 1% PtdIns(3)P, and Rab5(Q79L) show that all proteins are effectively incorporated into the membrane upon reconstitution.

(c) Related to Fig. 2a. Representative confocal microscopy images of HeLa cells fixed 5 min after injection of 4-EE-SNARE liposomes with various concentration of PtdIns(3)P or PtdIns(5)P. Cells were immunolabeled for organelle markers or internalized Alexa Fluor 488-labeled Transferrin (Tfn). Scale bar, 5 µm.

(d) Colocalization between injected liposomes containing PtdIns(3)P or PtdIns(5)P with endogenous markers. All values were normalized to the degree of colocalization observed in control injections using protein-free liposomes without any PtdInsPx. The data represent mean values ± SEM of 3-9 independent experiments, with each individual measurement being represented by a black dot. at least 100 injected vesicles were analyzed for the colocalization with each organelle marker in each experiment. The data were evaluated by 1-way ANOVA with the Tukey multiple comparison test. No significant colocalization was observed.

(e) A cytosolic fraction obtained from HeLa cells was incubated with either 0% or 1% PtdIns(3)P containing liposomes reconstituted with 4-EE-SNAREs. Subsequently, unbound proteins were separated using a flotation gradient. The liposome fraction was then analyzed by immunoblotting for Vps51, showing that binding of Vps51 is not dependent on PtdIns(3)P.

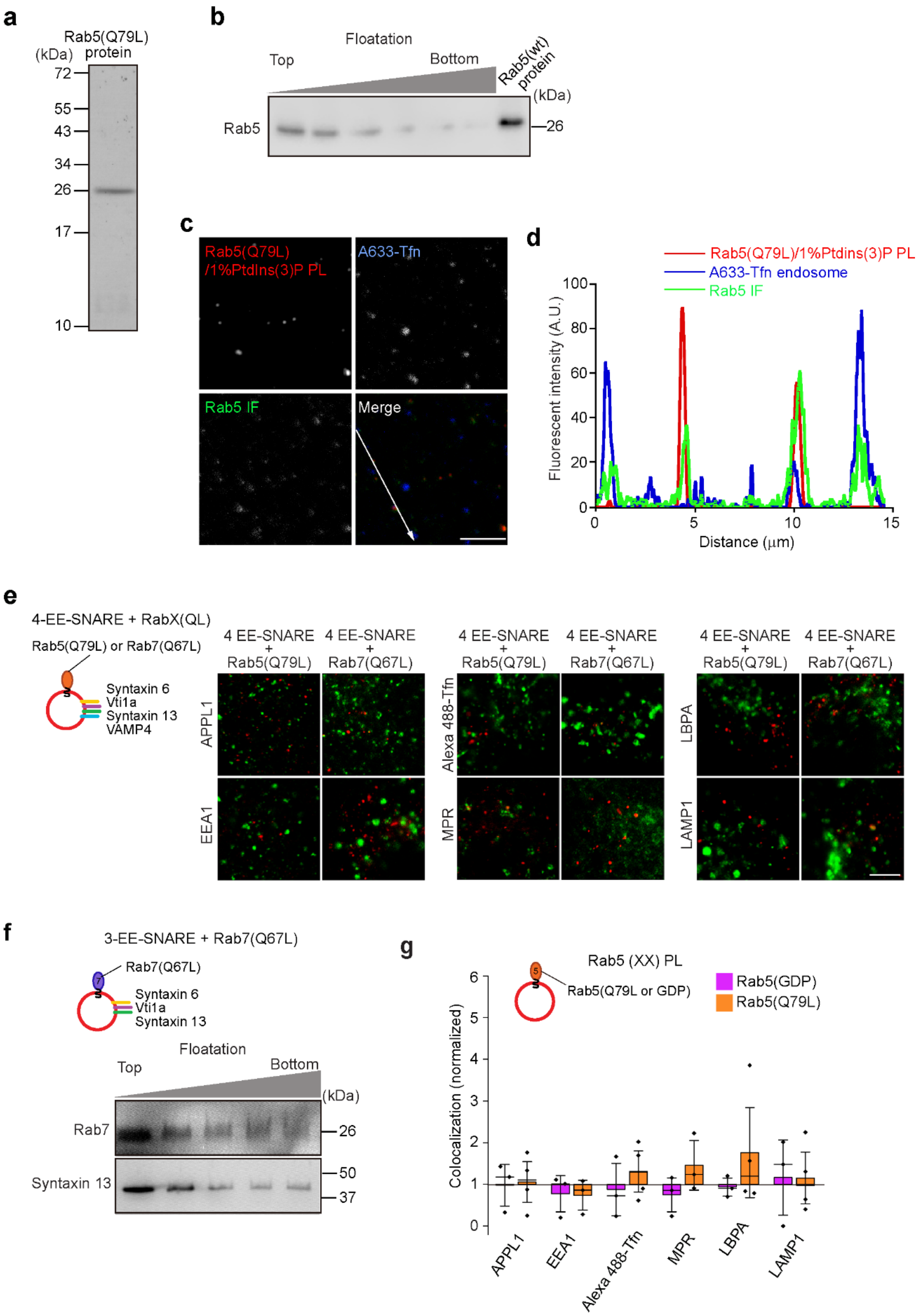
a
Rab5(Q79L) protein
(kDa)
72
55
43
34
26
17
10
b
Floatation
Top
Bottom
Rab5(wt) protein
(kDa)
Rab5
26
c
Rab5(Q79L) /1%PtdIns(3)P PL
A633-Tfn
Rab5 IF
Merge
d
Rab5(Q79L)/1%Ptdins(3)P PL
A633-Tfn endosome
Rab5 IF
Fluorescent intensity (A.U.)
100
80
60
40
20
0
0
5
10
15
Distance (μm)
e
4-EE-SNARE + RabX(QL)
Rab5(Q79L) or Rab7(Q67L)
Syntaxin 6
Vti1a
Syntaxin 13
VAMP4
4 EE-SNARE + Rab5(Q79L)
4 EE-SNARE + Rab7(Q67L)
APPL1
EEA1
Alexa 488-Tfn
MPR
LBPA
LAMP1
f
3-EE-SNARE + Rab7(Q67L)
Rab7(Q67L)
Syntaxin 6
Vti1a
Syntaxin 13
Floatation
Top
Bottom
(kDa)
Rab7
26
Syntaxin 13
50
37
g
Rab5 (XX) PL
Rab5(Q79L or GDP)
Rab5(GDP)
Rab5(Q79L)
Colocalization (normalized)
6
5
4
3
2
1
0
APPL1
EEA1
Alexa 488-Tfn
MPR
LBPA
LAMP1

Supplementary Figure S3

(a) Coomassie brilliant blue staining of an SDS PAGE of purified prenylated-Rab5 (Q79L) protein expressed in Sf9 cells.

(b) Flotation gradients of liposomes containing endosomal lipids and reconstituted with purified wt-Rab5, confirming that the protein is quantitatively incorporated into liposomes.

(c-d) Comparison of Rab5 protein levels between injected Rab5(Q79L) reconstituted liposomes and endogenous endosomes. (c) To label early endosomes, Alexa Fluor 633-Transferrin (Tfn) was internalized for 10 min, and then Rab5(Q79L) proteoliposomes containing 1% PtdIns(3)P were microinjected followed by immunostaining (IF) with anti-Rab5 antibody. Scale bar, 5 µm. (d) An intensity plot of the line scan (a white line) in the pictures on (c).

(e) Related to Fig. 2c. Colocalization between organellar markers and injected liposomes containing the 4-EE-SNAREs and prenylated Rab5(Q79L) or prenylated Rab7(Q67L). Cells were immunolabeled for organelle markers. Scale bar, 5 µm.

(f) Flotation gradients of liposomes reconstituted with purified Rab7(Q67L) and 3-EE-SNAREs. Protein distribution was detected by immunoblotting using anti-Rab7 or anti-Syntaxin 13-specific antibodies.

(g) Colocalization between Rab5 (GDP) or Rab5 (Q79L) containing liposomes with endogenous markers. All values were normalized to the degree of colocalization observed in control injections using protein-free liposomes. The data represent mean values ± SEM of 3-4 independent experiments, with each individual measurement being represented by a black dot. at least 100 injected vesicles were analyzed for the colocalization with each organelle marker in each experiment. The data were evaluated by 1-way ANOVA with the Tukey multiple comparison test. No significant colocalization was observed.

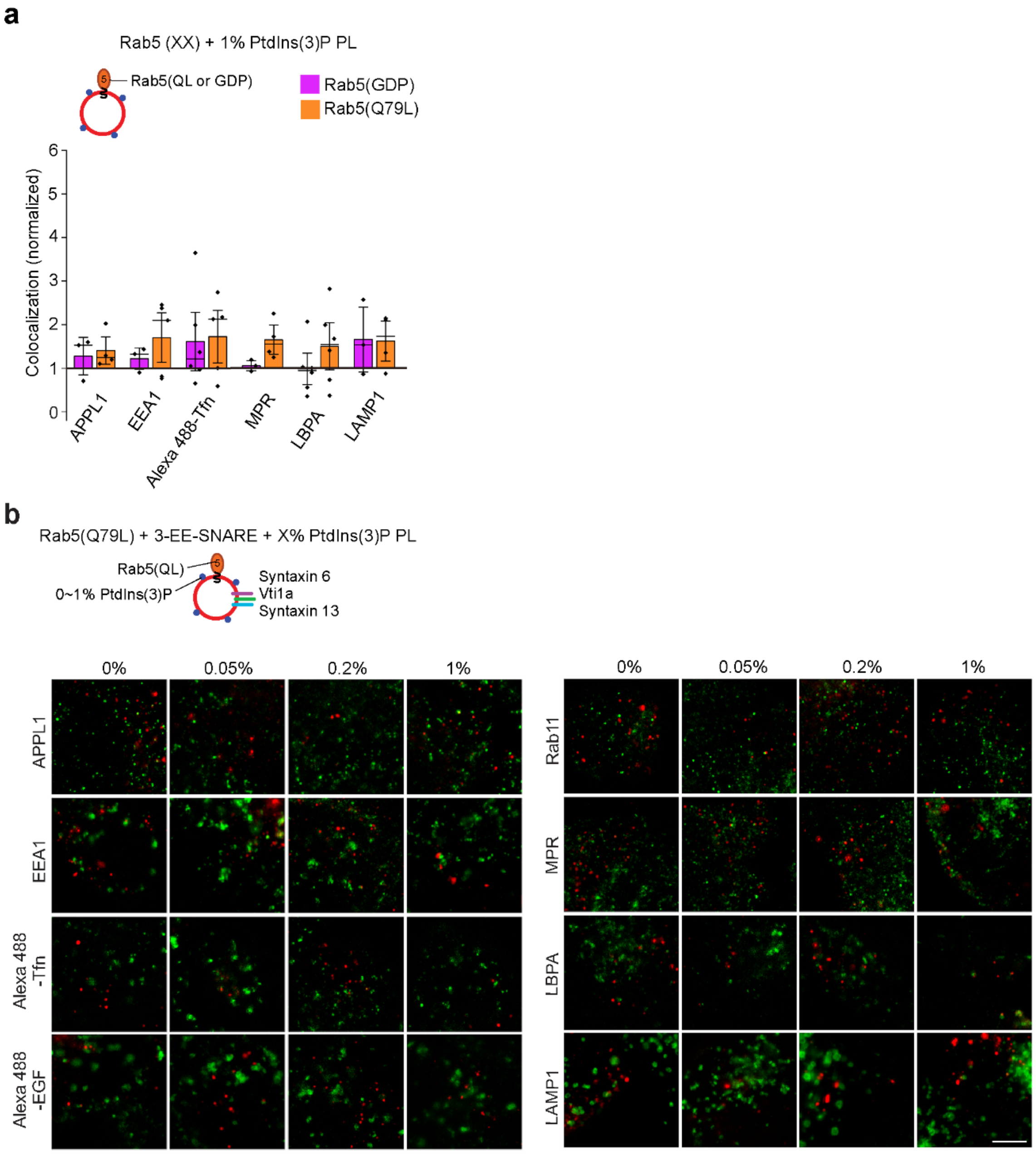

Supplementary Figure S4

(a) Colocalization between Rab5 (GDP) or Rab5 (Q79L) and 1% PtdIns(3)P containing liposomes with endogenous markers. All values were normalized to the degree of colocalization observed in control injections using protein-free liposomes. The data represent mean values ± SEM of 3-6 independent experiments, with each individual measurement being represented by a black dot. at least 100 injected vesicles were analyzed for the colocalization with each organelle marker in each experiment. The data were evaluated by 1-way ANOVA with the Tukey multiple comparison test. No significant colocalization was observed.

(b) Related to Fig. 3b. Representative confocal microscopy images of HeLa cells fixed 5 min after injection of 4-EE-SNARE and Rab5(Q79L) liposomes containing various concentration of PtdIns(3)P. Cells were immunolabeled for organelle markers. Scale bar, 5 µm.

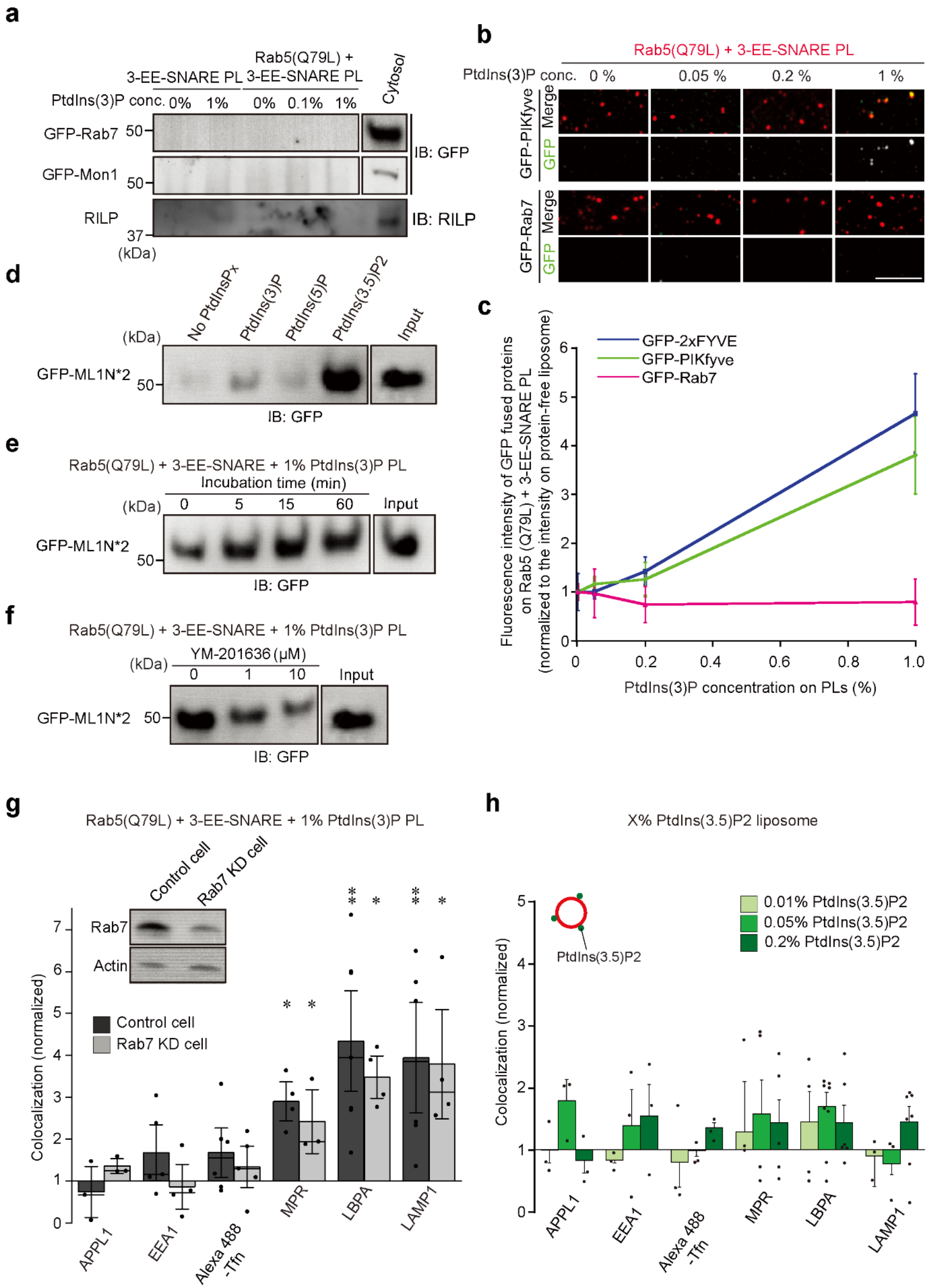

a
3-EE-SNARE PL
Rab5(Q79L) + 3-EE-SNARE PL
Cytosol
PtdIns(3)P conc. 0% 1% 0% 0.1% 1%
GFP-Rab7
GFP-Mon1
RILP
IB: GFP
IB: RILP
(kDa)
b
Rab5(Q79L) + 3-EE-SNARE PL
PtdIns(3)P conc. 0 % 0.05 % 0.2 % 1 %
GFP-PIKfyve
GFP-Rab7
Merge
GFP
c
GFP-2xFYVE
GFP-PIKfyve
GFP-Rab7
Fluorescence intensity of GFP fused proteins on Rab5 (Q79L) + 3-EE-SNARE PL (normalized to the intensity on protein-free liposome)
PtdIns(3)P concentration on PLs (%)
d
No PtdInsPx
PtdIns(3)P
PtdIns(5)P
PtdIns(3,5)P2
Input
GFP-ML1N*2
IB: GFP
e
Rab5(Q79L) + 3-EE-SNARE + 1% PtdIns(3)P PL
Incubation time (min)
0 5 15 60 Input
GFP-ML1N*2
IB: GFP
f
Rab5(Q79L) + 3-EE-SNARE + 1% PtdIns(3)P PL
YM-201636 (μM)
0 1 10 Input
GFP-ML1N*2
IB: GFP
g
Rab5(Q79L) + 3-EE-SNARE + 1% PtdIns(3)P PL
Control cell
Rab7 KD cell
Rab7
Actin
Colocalization (normalized)
APPL1
EEA1
Alexa 488 -Tfn
MPR
LBPA
LAMP1
h
X% PtdIns(3.5)P2 liposome
PtdIns(3.5)P2
0.01% PtdIns(3.5)P2
0.05% PtdIns(3.5)P2
0.2% PtdIns(3.5)P2
Colocalization (normalized)
APPL1
EEA1
Alexa 488 -Tfn
MPR
LBPA
LAMP1

Supplementary Figure S5

(a) Neither GFP-Rab7, GFP-Mon1 nor endogenous RILP was recruited to proteoliposomes reconstituted with early endosomal SNAREs and/or Rab5 (Q79L) and various combinations of phosphoinositides. The liposomes (all containing 1% Biotin-PE for subsequent isolation) were incubated with cytosolic extracts from HeLa cells expressing GFP-Rab7 or GFP-Mon1 for 30 min at 37°C. The proteoliposomes were precipitated with Streptavidin-conjugated beads, and recruited proteins were detected by immunoblotting with an anti-GFP antibody.
(b-c) Microscopy-based detection of protein recruitment to liposomes. Proteoliposomes reconstituted with EE-SNAREs, Rab5 (Q79L) and various combinations of phosphoinositides were incubated with a cytosolic fraction from HeLa cells overexpressing GFP-tagged proteins. The liposomes were then immobilized on coverslips, and the recruited proteins were detected by immunostaining with anti-GFP antibody. (b) Representative images of the recruitment of GFP-PIKfyve or GFP-Rab7. Scale bar, 5 µm. (c) Quantification of protein recruitment to the liposomal membrane. The fluorescence intensity of GFP on liposomes was calculated and normalized to the intensity of cytosol containing GFP. GFP-2xFYVE protein was used as a reporter for the concentration of PtdIns(3)P. The data show mean values ± SEM of 6 independent experiments.
(d-e) The recruitment of the GFP-ML1N*2 probe to liposomes was assessed by flotation of protein-liposome mixtures on Nycodenz gradients. (d) GFP-ML1N*2 specifically associated with PtdIns(3.5)P2. Liposomes with or without PtdInsPx were incubated with a GFP-ML1N*2 containing cytosol fraction for 5 min at 37°C. (e) PtdIns(3.5)P2 was produced in a time dependent fashion. Liposomes reconstituted with 3-EE-SNAREs, 1% PtdIns(3)P, and Rab5(Q79L) were incubated with GFP-ML1N*2 expressing cytosol fraction for various times at 37°C. (f) PtdIns(3.5)P2 conversion was inhibited by YM-201636 in a dose-dependent manner. Liposomes reconstituted with 3-EE-SNAREs, 1% PtdIns(3)P, and Rab5(Q79L) were incubated with GFP-ML1N*2 expressing cytosol fraction in presence of 1 µM or 10 µM YM-201636 for 10 min at 37°C.
(g) Targeting of liposomes reconstituted with 3-EE-SNAREs, 1% PtdIns(3)P, and Rab5(Q79L) in control or Rab7 knock-down HeLa cells. Insert shows an immunoblot for Rab7 in a knock-down (KD) cell line. Normalization was carried out using protein-free liposomes containing 1% PtdIns(3)P. The data represent mean values ± SEM of 3-6 independent experiments, with each individual measurement being represented by a black dot. At least 100 injected vesicles were analyzed for the colocalization with each organelle marker in each experiment. Significance was determined by 1-way ANOVA with the Tukey multiple comparison test. Stars indicate significance: * $P<0.05$, ** $P<0.01$.
(h) Colocalization between organelle markers and liposomes containing various concentration of PtdIns(3.5)P2. No significant colocalization with any of the tested organelle markers was observed. The data represent mean values ± SEM of 3-7 independent experiments, with each individual measurement being represented by a black dot. at least 100 injected vesicles were analyzed for the colocalization with each organelle marker in each experiment. The data were evaluated by 1-way ANOVA with the Tukey multiple comparison test.

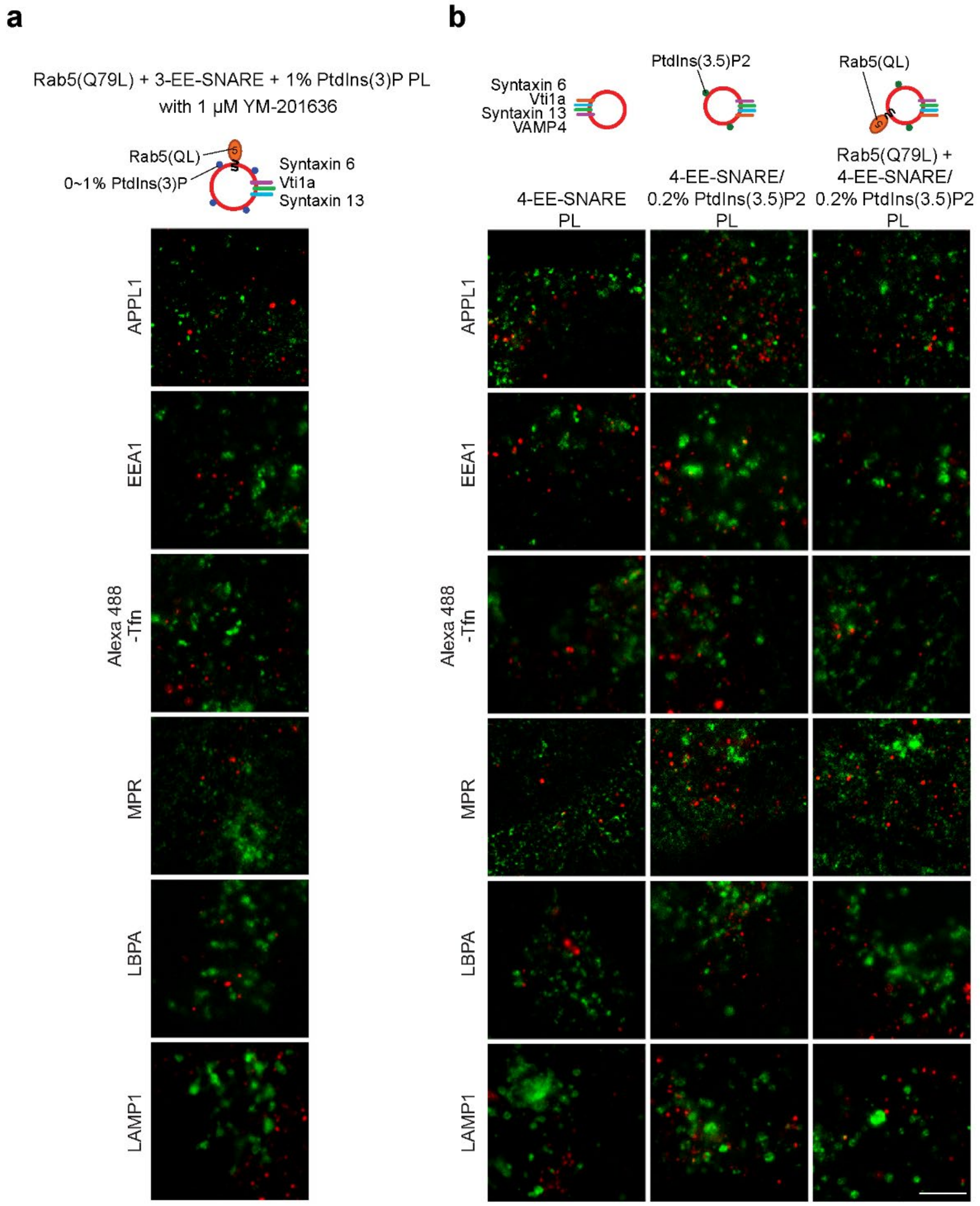

Supplementary Figure S6

(a) Related to Fig. 5c. Representative confocal microscopy images of HeLa cells fixed 5 min after injection of 3-EE-SNARE and Rab5(Q79L) liposomes with 1% PtdIns(3)P upon treatment with YM-201636, a PIKfyve inhibitor. Cells were immunolabeled for organelle markers. Scale bar, 5 µm.

(b) Related to Fig. 5d. Representative confocal microscopy images of HeLa cells fixed 5 min after injection of 3-EE-SNARE and/or Rab5(Q79L) liposomes with 0.2% PtdIns(3.5)P2. Cells were immunolabeled for organelle markers. Scale bar, 5 µm.
