# Supplementary material for: Rab GTPases and phosphoinositides fine-tune SNAREs dependent targeting specificity of intracellular vesicle traffic
Source: Nat Commun. 2024 Mar 20;15:2508. doi: 10.1038/s41467-024-46678-x (PMC10954720; doi:10.1038/s41467-024-46678-x)
Supplement: Supplementary file 3 — Reporting Summary [file 41467_2024_46678_MOESM3_ESM.pdf]

nature portfolio

Corresponding author(s): Reinhard jahn

Last updated by author(s): Feb 1, 2024

Reporting Summary

Nature Portfolio wishes to improve the reproducibility of the work that we publish. This form provides structure for consistency and transparency in reporting. For further information on Nature Portfolio policies, see our Editorial Policies and the Editorial Policy Checklist.

Statistics

For all statistical analyses, confirm that the following items are present in the figure legend, table legend, main text, or Methods section.

n/a	Confirmed	
☐	☒	The exact sample size (*n*) for each experimental group/condition, given as a discrete number and unit of measurement
☐	☒	A statement on whether measurements were taken from distinct samples or whether the same sample was measured repeatedly
☐	☒	The statistical test(s) used AND whether they are one- or two-sided<br>*Only common tests should be described solely by name; describe more complex techniques in the Methods section.*
☒	☐	A description of all covariates tested
☐	☒	A description of any assumptions or corrections, such as tests of normality and adjustment for multiple comparisons
☐	☒	A full description of the statistical parameters including central tendency (e.g. means) or other basic estimates (e.g. regression coefficient) AND variation (e.g. standard deviation) or associated estimates of uncertainty (e.g. confidence intervals)
☐	☒	For null hypothesis testing, the test statistic (e.g. *F*, *t*, *r*) with confidence intervals, effect sizes, degrees of freedom and *P* value noted<br>*Give P values as exact values whenever suitable.*
☒	☐	For Bayesian analysis, information on the choice of priors and Markov chain Monte Carlo settings
☒	☐	For hierarchical and complex designs, identification of the appropriate level for tests and full reporting of outcomes
☒	☐	Estimates of effect sizes (e.g. Cohen's *d*, Pearson's *r*), indicating how they were calculated

Our web collection on statistics for biologists contains articles on many of the points above.

Software and code

Policy information about availability of computer code

Data collection	All the images with microscopy were collected with Zen software (Carl Zeiss)<br>Western blot images were collected with LAS-1000 (FujiFilm)
Data analysis	Colocalization between injected liposomes and endogenous organelles were analyzed with a algorithm provided by Prof. Silvio Rizzoli. Fluorescence intensity and perinuclear index was analyzed with Image J software.

For manuscripts utilizing custom algorithms or software that are central to the research but not yet described in published literature, software must be made available to editors and reviewers. We strongly encourage code deposition in a community repository (e.g. GitHub). See the Nature Portfolio guidelines for submitting code & software for further information.

Data

Policy information about availability of data

All manuscripts must include a data availability statement. This statement should provide the following information, where applicable:

- Accession codes, unique identifiers, or web links for publicly available datasets
- A description of any restrictions on data availability
- For clinical datasets or third party data, please ensure that the statement adheres to our policy

The plasmids and experimentally data that support the findings of this study are available from the corresponding authors upon reasonable request.

Human research participants

Policy information about studies involving human research participants and Sex and Gender in Research.

Reporting on sex and gender	N/A
Population characteristics	N/A
Recruitment	N/A
Ethics oversight	N/A

Note that full information on the approval of the study protocol must also be provided in the manuscript.

Field-specific reporting

Please select the one below that is the best fit for your research. If you are not sure, read the appropriate sections before making your selection.

☒ Life sciences ☐ Behavioural & social sciences ☐ Ecological, evolutionary & environmental sciences

For a reference copy of the document with all sections, see nature.com/documents/nr-reporting-summary-flat.pdf

Life sciences study design

All studies must disclose on these points even when the disclosure is negative.

Sample size	The number of independent experiments used for the calculation of mean values and S.E.M. values is given in the legend to each panel. No statistical methods were applied to predetermine sample size.
Data exclusions	No data were excluded from analysis.
Replication	Experiments were repeated at least three independent experiments. If there were outliers, experiments were performed more to reduce S.E.M..
Randomization	no applicable
Blinding	Staining and analysis were performed blinded.

Reporting for specific materials, systems and methods

We require information from authors about some types of materials, experimental systems and methods used in many studies. Here, indicate whether each material, system or method listed is relevant to your study. If you are not sure if a list item applies to your research, read the appropriate section before selecting a response.

Materials & experimental systems

n/a	Involved in the study	
☐	☒	Antibodies
☐	☒	Eukaryotic cell lines
☒	☐	Palaeontology and archaeology
☒	☐	Animals and other organisms
☒	☐	Clinical data
☒	☐	Dual use research of concern

Methods

n/a	Involved in the study	
☒	☐	ChIP-seq
☒	☐	Flow cytometry
☒	☐	MRI-based neuroimaging

Antibodies

Antibodies used	Primary Antibodies<br>Anti-APPL1 (Rabbit monoclonal, #3858, Cell Signaling Technology)<br>anti-Rab7 (Rabbit monoclonal, #9367, Cell Signaling Technology)<br>anti-EEA1 (Mouse monoclonal, clone 14, #612006, Becton, Dickinson)<br>anti-M6PR (Mouse monoclonal, clone 2G11, ab2733, Abcam)<br>anti-RILP (Rabbit monoclonal, ab140188, Abcam)

anti-LAMP1 (Rabbit polyclonal, ab24170, Abcam)
anti-PIKfyve (Rabbit monoclonal, AF7885, R&D systems)
anti-LBPA (Mouse monoclonal, clone 6C4, Z-PLBPA, Echelon)
anti-Golgin97 (Mouse monoclonal, clone CDF4, A-21270, Thermo Fisher Scientific)
anti-Hrs (Rabbit polyclonal, PA5-27491, Thermo Fisher Scientific)
anti-Rab11 (Rabbit polyclonal, 71-5300, Thermo Fisher Scientific)
anti-Rabenosyn-5 (Rabbit polyclonal, 22228-1-AP, Proteintech)
anti-Rabankyrin5 (Rabbit polyclonal, 11321, Sigma-Aldrich)
anti-GFP (Rabbit polyclonal, 132002, Synaptic system)
anti-VPS51 (Rabbit polyclonal, HPA039650, Sigma-Aldrich)
anti-beta-actin (Rabbit polyclonal, 251003, Synaptic Systems)
anti-Rab5 (Mouse Monoclonal, clone 621.3, 108011, Synaptic Systems)
anti-syntaxin 6 (Rabbit polyclonal, 110062, Synaptic System)
anti-syntaxin 13 (Rabbit polyclonal, 110132, Synaptic System)
anti-Vti1a (Rabbit polyclonal, 165003, Synaptic System)
anti-VAMP4 (Rabbit polyclonal, 136002, Synaptic System)
anti-syntaxin 7 (Rabbit polyclonal, 110072, Synaptic System)
anti-syntaxin 8 (Rabbit polyclonal, 110083, Synaptic System)
anti-Vti1b (Rabbit polyclonal, 164002, Synaptic System)
anti-VAMP8 (Rabbit polyclonal, 104302, Synaptic System)

Secondary antibodies
Alexa Fluor 488-conjugated goat anti-mouse (115-545-166, Jackson ImmunoResarch Laboratories)
Cy3-conjugated goat anti-mouse (115-165-146, Jackson ImmunoResarch Laboratories)
Cy5-conjugated goat anti-mouse (115-175-166, Jackson ImmunoResarch Laboratories)
HRP-conjugated goat anti-mouse (STAR117P, BioRad)
Alexa Fluor 488-conjugated goat anti-rabbit (111-545-144, Jackson ImmunoResarch Laboratories)
Cy3-conjugated goat anti-rabbit (111-165-144, Jackson ImmunoResarch Laboratories)
Cy5-conjugated goat anti-rabbit (111-175-144, Jackson ImmunoResarch Laboratories)
HRP-conjugated goat anti-rabbit (5196-2504, BioRad)

Validation

Anti-APPL1 (Rabbit polyclonal, #3858, Cell Signaling Technology,https://www.cstj.co.jp/search/index.php?queryString=appl1&x=0&y=0)
anti-Rab7 (#9367, Cell Signaling Technology, https://www.cstj.co.jp/products/9367.html)
anti-EEA1 (#612006, Becton, Dickinson, https://www5.bdj.co.jp/reagents/view/610456)
anti-M6PR (ab2733, Abcam, https://www.abcam.co.jp/m6pr-cation-independent-antibody-2g11-ab2733.html)
anti-RILP (ab140188, Abcam, https://www.abcam.com/rilp-antibody-ab140188.html)
anti-LAMP1 (ab24170, Abcam, https://www.abcam.co.jp/lamp1-antibody-lysosome-marker-ab24170.html)
anti-PIKfyve (AF7885, R&D systems, https://www.rndsystems.com/products/human-pikfyve-antibody_af7885)
anti-LBPA (Z-PLBPA, Echelon, http://www.echelon-inc.com/index.php?module=Products&func=detail&id=767)
anti-Golgin97 (A-21270, Thermo Fisher Scientific, https://www.thermofisher.com/antibody/product/Golgin-97-Antibody-clone/CDF4-Monoclonal/A-21270)
anti-Hrs (PA5-27491, Thermo Fisher Scientific, https://www.thermofisher.com/antibody/product/HGS-Antibody-Polyclonal/PA5-27491)
anti-Rab11 (Rabbit polyclonal, 71-5300, Thermo Fisher Scientific, https://www.thermofisher.com/antibody/primary/target/rab11)
anti-Rabenosyn-5 (22218-1-AP, Proteintech, https://www.ptglab.com/products/ZFYVE20-Antibody-22218-1-AP.htm)
anti-Rabankyrin5 (11321, Sigma-Aldrich)
anti-GFP (132002, Synaptic system, https://www.sysy.com/products/gfp/facts-132002.php)
anti-VPS51 (Rabbit polyclonal, HPA039650, Sigma-Aldrich, https://www.sigmaaldrich.com/JP/ja/product/sigma/hpa039650)
anti-beta-actin (Rabbit polyclonal, 251003, Synaptic system, https://www.sysy.com/products/b-actin/facts-251003.php)
anti-Rab5 (108011, Synaptic system, https://www.sysy.com/products/rab5/facts-108011.php)
anti-syntaxin 6 (110062, Synaptic system, https://www.sysy.com/products/syntaxin6/facts-110062.php)
anti-Vti1a (165003, Synaptic system, https://sysy.com/product/165003)
anti-syntaxin 13 (110132, Synaptic system, https://www.sysy.com/products/syntaxin13/facts-110132.php)
anti-VAMP4 (136002, Synaptic system, https://www.sysy.com/products/vamp4/facts-136002.php)
anti-syntaxin 7 (110072, Synaptic system, https://sysy.com/product/110072#list)
anti-syntaxin 8 (110083, Synaptic system, https://sysy.com/product/110083#list)
anti-Vti1b (164002, Synaptic system, https://sysy.com/product/164002#list)
anti-VAMP8 (104302, Synaptic system, https://sysy.com/product/104302)

Eukaryotic cell lines

Policy information about cell lines and Sex and Gender in Research

Cell line source(s)

HeLa cells

Authentication

Cell line authentication was not performed.

Mycoplasma contamination

The cell lines were not tested for Mycoplasma contamination.

Commonly misidentified lines
(See ICLAC register)

No commonly misidentified cell lines used.
